# FCA-STNet: Spatiotemporal Growth Prediction and Phenotype Extraction from Image Sequences for Cotton Seedlings

**DOI:** 10.3390/plants14152394

**Published:** 2025-08-02

**Authors:** Yiping Wan, Bo Han, Pengyu Chu, Qiang Guo, Jingjing Zhang

**Affiliations:** 1 College of Computer and Information Engineering, Xinjiang Agricultural University, Urumqi 830052, China; 2 Engineering Research Center of Intelligent Agriculture Ministry of Education, Urumqi 830052, China; 3 Xinjiang Agricultural Informatization Engineering Technology Research Center, Urumqi 830052, China

**Keywords:** growth prediction, phenotype, cotton seedling, ST-LSTM, FCA

## Abstract

To address the limitations of the existing cotton seedling growth prediction methods in field environments, specifically, poor representation of spatiotemporal features and low visual fidelity in texture rendering, this paper proposes an algorithm for the prediction of cotton seedling growth from images based on FCA-STNet. The model leverages historical sequences of cotton seedling RGB images to generate an image of the predicted growth at time t + 1 and extracts 37 phenotypic traits from the predicted image. A novel STNet structure is designed to enhance the representation of spatiotemporal dependencies, while an Adaptive Fine-Grained Channel Attention (FCA) module is integrated to capture both global and local feature information. This attention mechanism focuses on individual cotton plants and their textural characteristics, effectively reducing the interference from common field-related challenges such as insufficient lighting, leaf fluttering, and wind disturbances. The experimental results demonstrate that the predicted images achieved an MSE of 0.0086, MAE of 0.0321, SSIM of 0.8339, and PSNR of 20.7011 on the test set, representing improvements of 2.27%, 0.31%, 4.73%, and 11.20%, respectively, over the baseline STNet. The method outperforms several mainstream spatiotemporal prediction models. Furthermore, the majority of the predicted phenotypic traits exhibited correlations with actual measurements with coefficients above 0.8, indicating high prediction accuracy. The proposed FCA-STNet model enables visually realistic prediction of cotton seedling growth in open-field conditions, offering a new perspective for research in growth prediction.

## 1. Introduction

With the integration of information technology into agriculture, the concept of “Digital Plant” has emerged, providing an important reference for plant growth monitoring. “Digital Plant” is a newly developed interdisciplinary field that tightly integrates plant science, computer technology, artificial intelligence, and scientific computing [1]. Crop growth image prediction offers the ability to generate images of the future crop growth at given time points, enabling more realistic and intuitive visualization of crop development. Specifically, developing methods for predicting cotton seedling growth can help elucidate the interactions between different growth stages and the environmental conditions, thereby serving as a theoretical tool that promotes the transition of cotton growth research from qualitative to quantitative analysis [2,3]. This is of great significance for growth monitoring and research on the breeding of cotton seedlings.

Traditional plant growth prediction methods typically rely on phenotypic information and empirical mathematical formulas to reconstruct the overall plant appearance and texture. For instance, Espana et al. [4] developed an empirical model of maize leaf growth, simulating the leaves as 2D rectangular planes and mapping them into a 3D space to generate a visual model of the maize leaves. Qian et al. [5] proposed a thermally driven, four-dimensional coupled model for maize growth, enabling simulation and visualization of the canopy’s structural parameters. However, such approaches heavily depend on subjective expertise, suffer from limited generalizability, and lack the complexity to capture plant growth diversity, thereby restricting the realism and visualization quality of the resulting digital plant images.

Recent advancements in optical imaging, data storage, and information technology have significantly enhanced computers’ image processing capabilities, which are vital for plant phenotype prediction [6,7]. For example, Kim et al. [8,9] applied spatial transformer networks (STNs) to model the dynamic leaf growth by learning the color and structural changes in plant images, achieving accurate predictions for small plant leaves, though these methods struggle when applied to structurally complex plants. Sakurai et al. [10] proposed a convolutional LSTM-based framework that predicts future plant images from past image sequences. Wang Chunying et al. [11] used an ST-LSTM model to forecast the top-view growth of *Arabidopsis thaliana* and further incorporated memory-in-memory (MIM) networks to predict the side-view growth of wheat [12]. Yasrab et al. [13] trained the FutureGAN model to perform predictive segmentation of plant leaves and roots at future time intervals. Drees et al. [14] applied a Pix2Pix-based network to predict the top-view growth of rapeseed under varying water and fertilizer conditions. Duan Lingfeng et al. [15] utilized an improved Pix2Pix-HD model to predict rice growth at the grain-filling stage using panicle emergence images through a data-driven approach.

Although deep learning has yielded encouraging progress in plant growth prediction, studies focused on cotton seedlings remain limited. Most current efforts concentrate on crops with simple structures or datasets from controlled greenhouse environments. Research into predicting morphologically complex and highly individual plants in natural field conditions is still lacking. The growth of cotton seedlings features both long-term dependencies and local variations, posing challenges for accurate spatiotemporal feature extraction. In open-field conditions, dynamic changes in the time of day and weather significantly affect image clarity due to inconsistent lighting [16]. Additionally, wind-induced motion and the reflection of light from wet soil during irrigation may blur plant contours and obscure texture boundaries.

To address the challenges discussed above, this study presents the following contributions:

1. Dataset Construction: Due to the lack of publicly available time-series image datasets of cotton seedling growth under real field conditions and suited to morphological similarity recognition tasks, we manually collected image sequences and applied data augmentation techniques to expand the dataset, thereby improving the generalization capability of the model.
2. FCA-STNet Architecture: We propose FCA-STNet, a prediction network based on RGB image sequences of cotton seedlings. The network is built upon a self-designed STNet backbone, which is tailored to enhance spatiotemporal feature extraction under conditions where long-term dependencies and local variations coexist. An Adaptive Fine-Grained Channel Attention (FCA) module is incorporated to dynamically reweight channel-wise features, suppressing the interference from non-uniform lighting and wind disturbances. This improves the clarity and robustness of feature representation and enables the model to better adapt to complex visual variations during cotton seedling growth.
3. Phenotypic Prediction and Evaluation: The predicted image at time t + 1 is used to extract 37 phenotypic traits, including the color, morphology, and texture features. These traits serve both as a basis for evaluating the model accuracy and as a visual reference for assessing the cotton seedling status at t + 1, thereby supporting the optimization of agronomic practices.

## 2. Results

In this study, the FCA-STNet model was adopted. The RGB cotton seedling images taken at the first two time points were input to generate images of the predicted growth at the future time point t + 1. With this as the goal, 32 sets of test set sequences were constructed, and a total of 32 images predicting the growth at the future time point t + 1 were obtained. From each predicted image, 37 phenotypic features were extracted, and Pearson correlation coefficient analysis was conducted with the corresponding real images to evaluate the phenotypic consistency of the prediction results.

The experiment was completed on a rented workstation from the AutoDL platform (https://www.autodl.com), a cloud computing service based in China.The workstation had the following specific configuration: a 16-core AMD EPYC 9K84 processor (with a total of 32 threads), 512 GB of memory, and an NVIDIA H20-NVLink graphics card (with 96 GB of video memory). The operating system was Ubuntu 22.04. The development environment was based on Anaconda, using PyTorch 2.1.0 and Python 3.10, along with CUDA 12.1 for GPU-accelerated training. During the training process, the Adam optimizer was adopted, with the learning rate set at 0.0001, the batch size at 32, and the total number of training rounds at 50 epochs.

All the evaluation indicators were calculated for the test set of 32 groups of cotton seedling image sequences, and the average values of each indicator were taken. The assessment method was based on the indicators defined in Section 4.1.3.

### 2.1. Dataset Construction and Preprocessing

**Figure 1:**
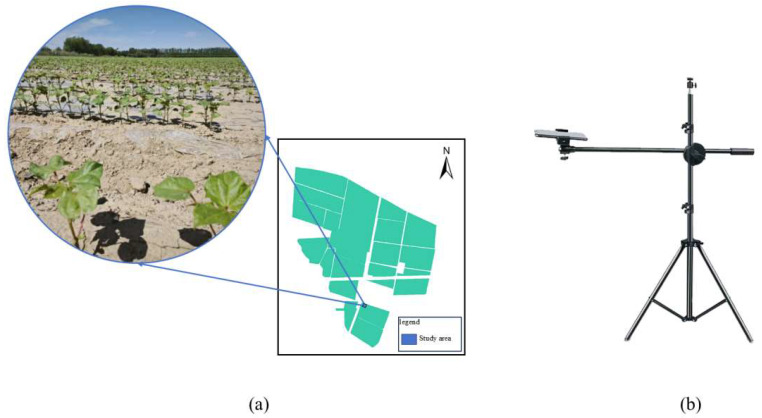
Experimental site and imaging equipment. (**a**) Field location; (**b**) imaging setup.

Time-series images of cotton seedlings were collected at Huaxing Farm, Daxiqu Town, Changji City, Xinjiang Uygur Autonomous Region (longitude of 87°29′ E, latitude of 44°22′ N), which has a temperate continental climate. The specific experimental location at Huaxing Farm is shown in Figure 1a. The cotton variety used was H33-1-4. To ensure consistent camera angles across the image sequences, a Xiaomi smartphone(Xiaomi Corporation, Sichuan, China) mounted on a fixed shooting frame was employed, as shown in Figure 1b. Between 23 May and 25 May 2024, every 24 h at around 9 a.m. every day, images were collected on the same farmland, obtaining 52 RGB image sequences of the top views of cotton seedlings. Each sequence contained three consecutive frames, and the original resolution of the images was 3072 pixels in height and 4096 pixels in width.

**Figure 2:**
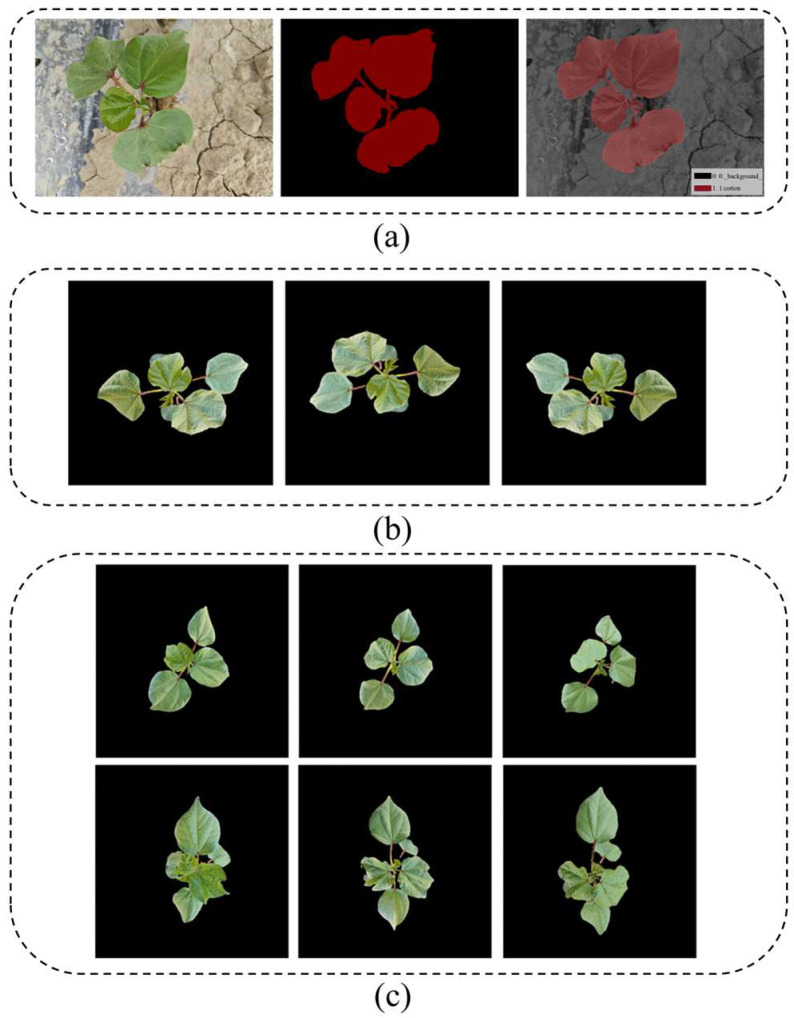
Cotton seedling dataset. (**a**) Visualization of dataset annotation: original image, label, and label overlay; (**b**) data augmentation: original, rotated 180°, and horizontally flipped; (**c**) image sequences with background removed: two examples shown in separate rows.

Image noise significantly affects the accuracy of cotton seedling growth prediction and must be minimized through preprocessing. In this study, a UNet segmentation model [17] was employed (Figure 2a). A dataset of 300 manually annotated cotton images was used to train the UNet model via transfer learning. The trained model was then applied to the cotton seedling image sequences to generate plant masks, effectively removing background noise. The resulting background-free images were resized to 256 pixels in height and 256 pixels in width. To improve the model’s generalization ability, data augmentation was performed through 180° rotation and horizontal flipping, as shown in Figure 2b [18]. This process yielded a dataset containing 156 sequences, with each sequence comprising 3 images, resulting in a total of 468 cotton seedling images for the prediction tasks. The dataset was randomly divided into a training set and a test set in an 8:2 ratio; that is, the training set had 124 sequences and the test set had 32 sequences. Each sequence in both sets included three consecutive images, with the first two used as the input and the third as the prediction target. Examples of the preprocessed sequences are shown in Figure 2c.

### 2.2. Prediction Results for Cotton Seedlings

#### 2.2.1. Comparison with Other Prediction Models

**Figure 3:**
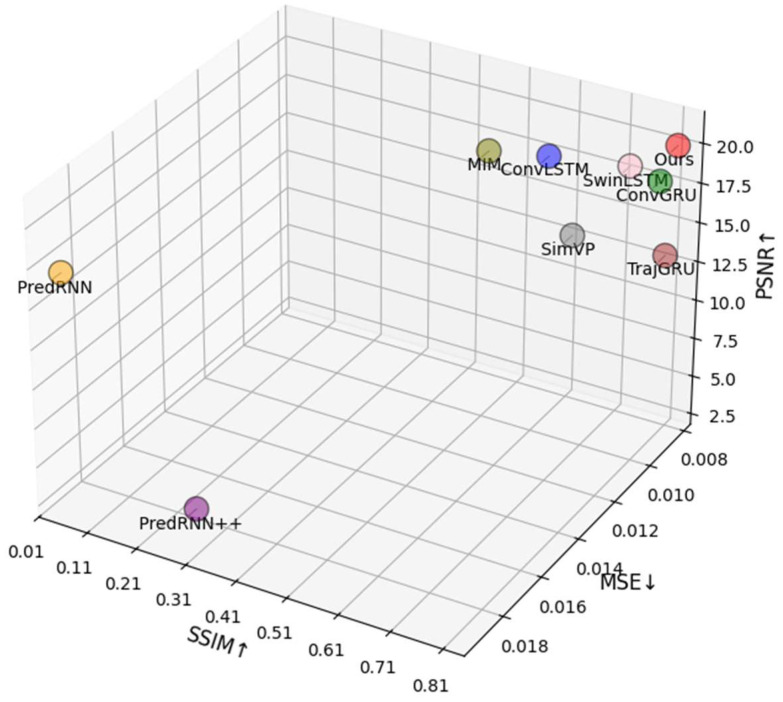
Comparison of different prediction models.

To further verify the effectiveness of FCA-STNet, we compared it with several benchmark models: ConvLSTM, ConvGRU, MIM [19], PredRNN [20], PredRNN++ [21], TrajGRU [22], SwinLSTM [23], and SimVP. Quantitative results are presented in Table 1, with a 3D visual comparison in Figure 3, where SSIM, MSE, and PSNR are mapped to the x, y, and z axes, respectively. Higher values indicate better performance.

FCA-STNet, represented by the red circle in Figure 3, achieved the best results across all four metrics: the SSIM (0.8339), PSNR (20.7011), MSE (0.0086), and MAE (0.0321). Comparison with PredRNN/PredRNN++/MIM: FCA-STNet significantly improved the realism of the morphological features. Comparison with ConvLSTM: In our model, the SSIM and PSNR improved by 38.1% and 11.3%, respectively. Although ConvLSTM captures spatiotemporal features via convolution, its single-layer structure limits long-term sequence modeling. Comparison with SwinLSTM: The MSE and MAE in our model decreased by 13.1% and 11.1%, while the SSIM and PSNR improved by 5.9% and 1.8%. SwinLSTM uses a hierarchical attention mechanism but suffers from high computational complexity and weak feature robustness. Comparison with TrajGRU: In our model, the MSE and MAE were reduced by 2.4% and 2.5%, with the SSIM and PSNR increasing by 2.8% and 55.5%, respectively. Though TrajGRU excels in dynamic object tracking, it struggles with fine-grained static detail recovery. Comparison with SimVP: The MSE and MAE dropped by 37.2% and 42.1% in our model, while the SSIM and PSNR rose by 2.8% and 2.5%. SimVP’s lightweight design limits its feature extraction depth and representational power.

These findings demonstrate that FCA-STNet effectively reduces information loss during feature extraction and sequence prediction. The integration of STNet and FCA enhances the network’s ability to capture both global and local features, especially textures and edges, thus improving the model’s visual fidelity and structural preservation.

#### 2.2.2. Ablation Study

**Figure 4:**
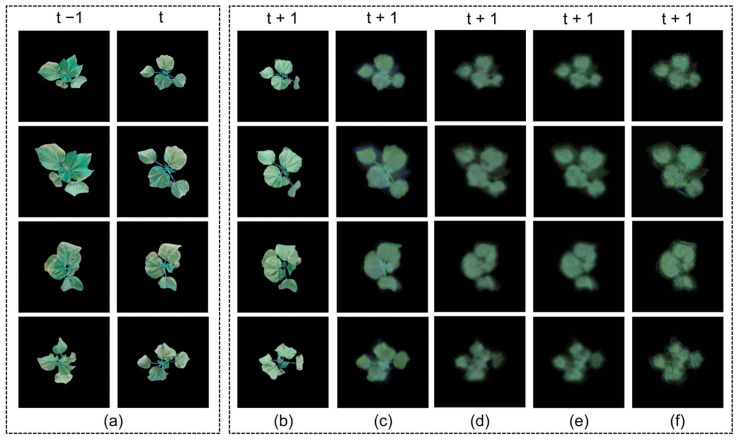
Growth prediction results from the ablation study. (**a**) Input sequence; (**b**) ground truth; (**c**) prediction by FCA-STNet; (**d**) prediction by STNet; (**e**) prediction with FCA added only after the Encoder; (**f**) prediction with FCA added only after the Translator.

**Figure 5:**
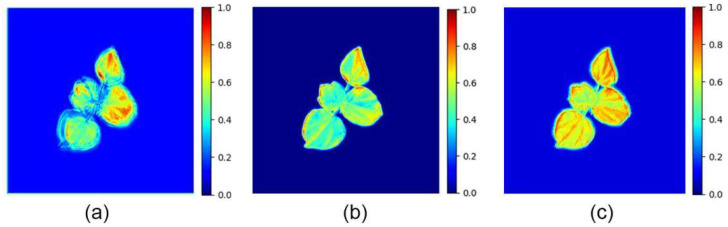
Heatmaps of feature extraction in ablation experiments. (**a**) FCA added only after Encoder; (**b**) FCA added only after Translator; (**c**) FCA-STNet.

To evaluate the contributions of different components, we conducted ablation studies using STNet as the baseline and selectively adding FCA modules at different positions. The results are shown in Table 2, and the corresponding feature extraction heatmaps and prediction results are shown in Figure 4 and Figure 5, respectively. The real and predicted time points were both the same: t − 1 was 23 May 2024, t was 24 May 2024, and t + 1 was 25 May 2024.

As seen in Figure 4, STNet’s predicted leaf shapes were discernible but suffered from blurry contours and a loss of detail. The model with FCA only after the Encoder improved the detail slightly, while the configuration with FCA only after the Translator produced blurred leaf outlines. FCA-STNet yielded the most visually accurate predictions, with sharp edges, well-defined contours, and clear leaf vein structures closely resembling the ground truth.

Adding FCA only after the Translator led to a performance drop, with the SSIM and PSNR decreasing by 3.24% and 14.98%, respectively. Adding FCA only after the Encoder yielded modest improvements. In contrast, FCA-STNet—with FCA modules added after both the Encoder and Translator—achieved improvements in the MSE (↓2.27%), MAE (↓0.31%), SSIM (↑4.73%, from 0.7962 to 0.8339), and PSNR (↑11.20%, from 18.6170 to 20.7011), confirming the effectiveness of the proposed design in real field conditions.

#### 2.2.3. Spatiotemporal Feature Extraction in Translator

In FCA-STNet, the Translator module uses two layers of ST-LSTM with 64 hidden dimensions to extract spatiotemporal features from cotton seedling sequences. To validate this design, we conducted ablation experiments varying the number of both the layers and hidden dimensions. Additionally, we compared ST-LSTM with other recurrent modules such as ConvLSTM and ConvGRU [24] to assess their effectiveness in capturing the spatiotemporal dynamics.

**Figure 6:**
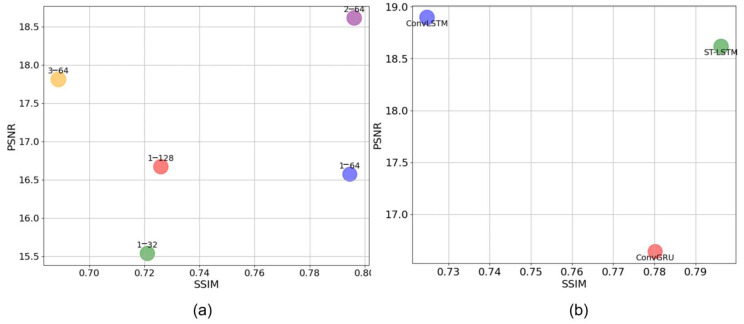
Comparison of SSIM and PSNR of different Translators. (**a**) Comparison of different layers and different hidden_dims parameter settings; (**b**) comparison of different spatiotemporal prediction models.

Table 3 and Figure 6 summarize the results. As shown in Figure 6a, the configuration with one ST-LSTM layer and 64 hidden dimensions achieved significant improvements across all the metrics, especially in the SSIM (0.7946) and PSNR (16.5742). Increasing the layer count to two further enhanced the SSIM by 0.2% and the PSNR by 12.37%, indicating improved modeling of the temporal dependencies. However, adding a third layer led to a decline in the SSIM and PSNR, likely due to overfitting and noise amplification.

As illustrated in Figure 6b, ST-LSTM outperformed ConvLSTM and ConvGRU in terms of the SSIM by 10.02% and 2.05%, respectively, suggesting superior texture detail learning. Although ConvLSTM achieved a marginally higher PSNR (1.5% above that of ST-LSTM), the difference was not substantial. These results confirm the effectiveness of ST-LSTM and the optimal configuration of FCA-STNet for modeling cotton seedling growth in real field conditions.

#### 2.2.4. Comparison of Attention Mechanisms

The FCA mechanism was introduced into the model at two distinct stages: after the Encoder and after the Translator. To evaluate the superiority of the FCA mechanism, comparative experiments were conducted with CGA [25], FSAS [26], and EPA [27] mechanisms. These attention modules were inserted at the same positions for a fair comparison. Detailed experimental results are shown in Table 4. It can be observed that the FCA mechanism achieved SSIM and PSNR scores of 0.8339 and 20.7011, respectively—higher than those achieved by the other attention mechanisms.

**Figure 7:**
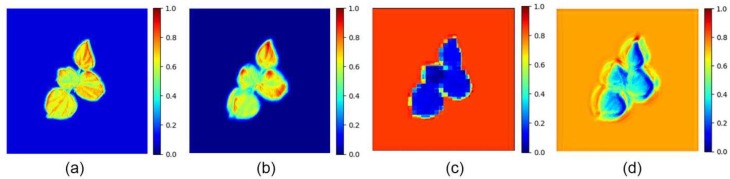
Heatmaps generated by different attention mechanisms. (**a**) FCA; (**b**) CGA; (**c**) FSAS; (**d**) EPA.

In addition, heatmaps were employed to compare the capability of each attention mechanism to extract cotton-specific features. The visualization results are presented in Figure 7. As shown in the figure, the FCA mechanism exhibited the best phenotype representation, offering more comprehensive feature extraction of cotton textures and edges. In contrast, the FSAS and EPA mechanisms tended to focus more on the background rather than the cotton itself.

### 2.3. Phenotypic Feature Correlation Analysis

**Figure 8:**
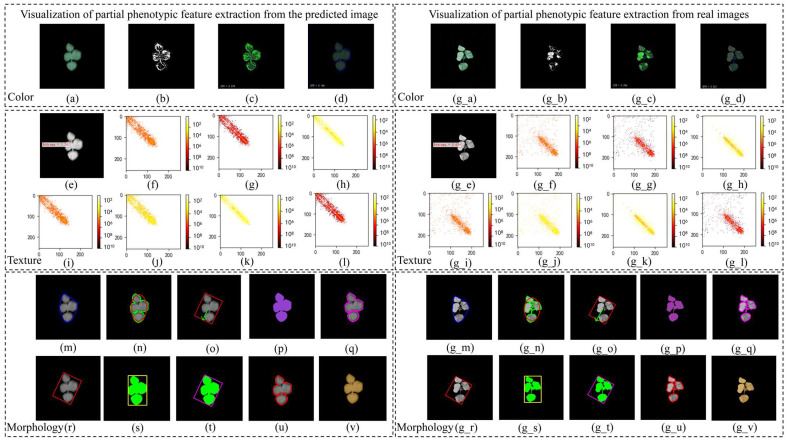
Diagram showing visualization process for some phenotypic features. (**a**) Original predicted image; (**b**) GPA; (**c**) GTR; (**d**) BTR; (**e**) E; (**f**) T1:0.9913; (**g**) T2:0.9138; (**h**) T3:12.6327; (**i**) T4:0.8709; (**j**) T11:1.7623; (**k**) T12:12.6327; (**l**) T13:0.9138; (**m**) A_HA; (**n**) R; (**o**) AN; (**p**) A_A; (**q**) A_P; (**r**) A_MBA; (**s**) A_TBR; (**t**) A_TMBR; (**u**) A_PAR; (**v**) TPA; (**g_a**) original true image; (**g_b**) GPA; (**g_c**) GTR; (**g_d**) BTR; (**g_e**) E; (**g_f**) T1:0.9570; (**g_g**) T2:0.9532; (**g_h**) T3:87.9004; (**g_i**) T4:0.9475; (**g_j**) T11:0.9015; (**g_k**) T12:87.9004; (**g_l**) T13:0.9532; (**g_m**) A_HA; (**g_n**) R; (**g_o**) AN; (**g_p**) A_A; (**g_q**) A_P; (**g_r**) A_MBA; (**g_s**) A_TBR; (**g_t**) A_TMBR; (**g_u**) A_PAR; (**g_v**) TPA.

**Figure 9:**
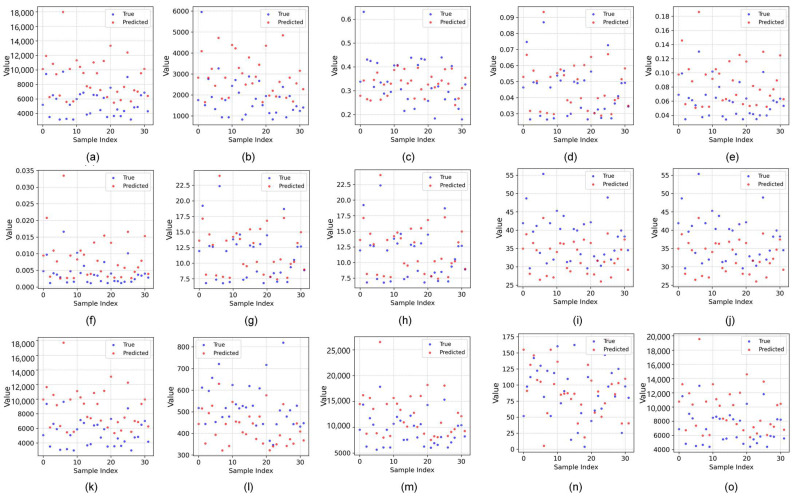
A scatter comparison chart of some phenotypic features extracted from the prediction graphs and real graphs of all the test sets. (**a**) TPA; (**b**) GPA; (**c**) GTR; (**d**) M; (**e**) SE; (**f**) S; (**g**) T7; (**h**) T8; (**i**) T14; (**j**) T15; (**k**) A_A; (**l**) A_P; (**m**) A_MBA; (**n**) AN; (**o**) A_HA.

**Figure 10:**
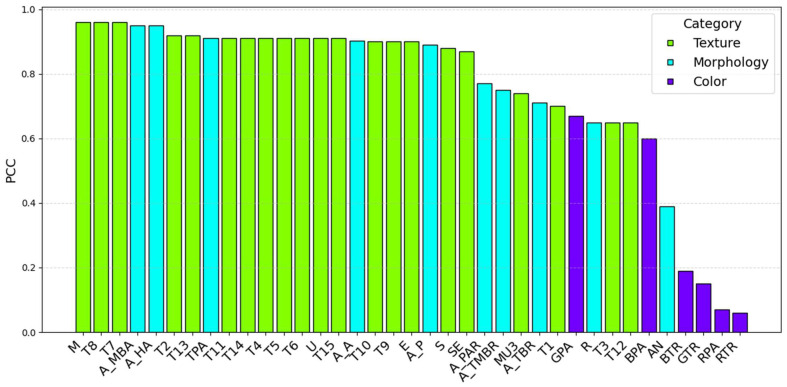
Comparison of different phenotypic characteristics.

Thirty-seven phenotypic features were extracted from the predicted images and real images in the test set using OpenCV. Figure 8 shows a visualization of the extraction of some phenotypic features. Figure 9 shows a scatter comparison chart displaying the results of a comparison between the predicted values and true values of all the test set samples for the extraction of some phenotypic features. The Pearson correlation coefficient was calculated to evaluate the prediction accuracy, as shown in Table 5 and Figure 10.

Most traits exhibited correlations with coefficients above 0.8, indicating strong overall predictive performance. For texture features, metrics such as the M (0.96), SE (0.87), S (0.88), U (0.91), E (0.90), T2 (0.92), T4 (0.91), T7/T8 (0.96), T9/T10 (0.90), T11 (0.91), and T13/T15 (0.92/0.91) all had strong correlations. This suggests that the model accurately captured localized dynamic features. A few features such as MU3, T3, and T12 performed slightly worse, likely due to the high variability in high-contrast regions. For morphological traits, metrics like the TPA, A_HA, A_A, and A_MBA all exceeded 0.90, showing strong predictive accuracy for geometric characteristics. However, the R (0.65) and AN (0.39) had lower correlations, likely due to wind-induced motion and shape distortion, which increased the recognition difficulty. For color features, the correlations were relatively weak. In particular, red-channel features showed near-zero correlations, indicating significant deviations in the color component predictions. This may have stemmed from inconsistencies in the color space transformations or insufficient extraction of color information.

## 3. Discussion

**Figure 11:**
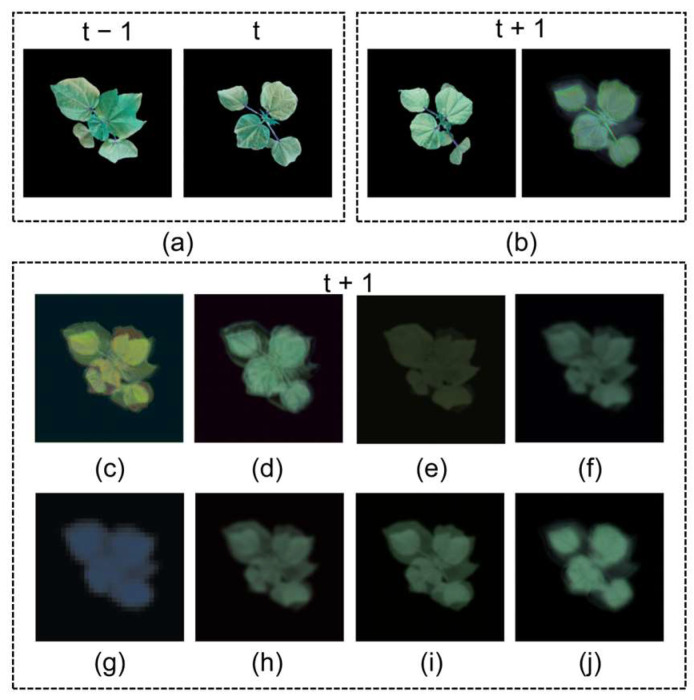
Prediction results of different models. (**a**) Input image sequence; (**b**) ground truth (left) and prediction by FCA-STNet (right); (**c**) PredRNN; (**d**) PredRNN++; (**e**) MIM; (**f**) ConvLSTM; (**g**) SwinLSTM; (**h**) ConvGRU; (**i**) TrajGRU; (**j**) SimVP.

**Figure 12:**
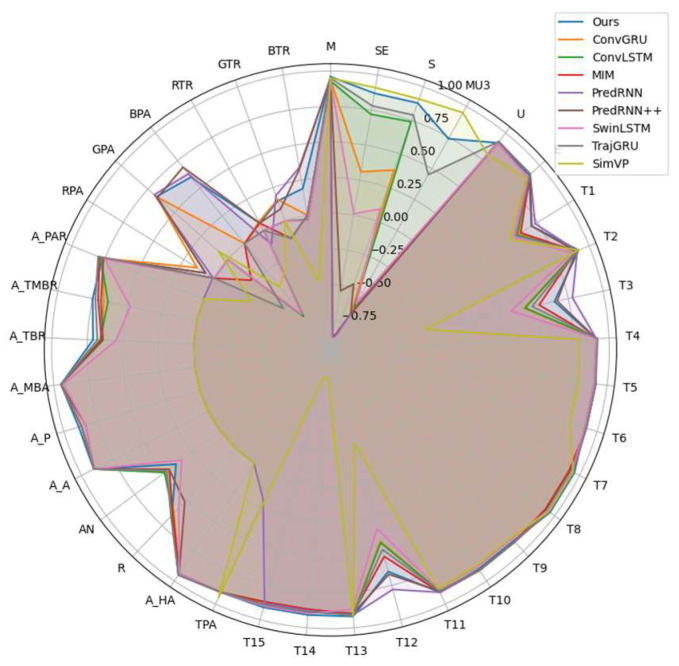
Radar plots of phenotype correlation coefficients across models.

The FCA-STNet model demonstrates the capability to effectively predict future images of cotton seedlings with complex morphological structures in open-field environments. Furthermore, it enables the extraction of phenotypic features—such as the texture, morphology, and color—from the predicted images. Based on both visual inspection and quantitative evaluation, the predicted images generated by FCA-STNet closely resembled real-world observations, exhibiting higher individual discriminability. Regarding the existing models, as illustrated in Figure 11 (predicted images generated by various models) and Figure 12 (radar plots of phenotype correlation coefficients), FCA-STNet consistently outperforms them across nearly all the types of feature predictions. It shows advantages in relation to its resolution, realism, visual similarity, individual differentiation, textural detail, and phenotypic information. Among the compared models, FCA-STNet delivers the most balanced and superior overall performance. These results indicate that the proposed approach provides accurate and reliable prediction of cotton seedling growth in field conditions.

Despite the promising performance of the proposed model in multiple aspects, there are still areas that require further exploration and refinement. This study represents a preliminary investigation into the prediction of seedling-stage cotton growth under field conditions. However, limitations exist, including a relatively small dataset and limited coverage of the full growth cycle. Future work could expand the prediction framework to multiple growth stages and incorporate larger and more diverse datasets to enhance the model robustness. Additionally, the present study only explored predictions for the next time step (t + 1), constrained by real-world factors such as environmental complexity, significant morphological variability, and the dynamic orientation of cotton leaves in the field. Extending the model’s predictive range to longer time horizons is a promising direction. While the model demonstrates some capabilities to extract color-related phenotypic features, its performance in this aspect remains suboptimal. Future studies could focus on enhancing the color feature extraction techniques. Overall, addressing these limitations and improving the model accordingly will facilitate more efficient and accurate prediction of cotton seedling growth in complex agricultural environments.

## 4. Materials and Methods

The materials were from the dataset described in Section 2.1. All the methods mentioned in the following text were used to test the model’s performance on this dataset.

### 4.1. FCA-STNet Network Architecture

In real-world field environments, cotton seedling growth is characterized by numerous subtle and continuous morphological changes, such as leaf expansion, plant height increases, and shifts in the leaf orientation. These environments exhibit high spatiotemporal complexity, with seedling development affected by the temperature, humidity, and light intensity—factors that vary nonlinearly across time and space. Additionally, the image quality is often degraded due to uneven illumination, motion blur, and inconsistent capture intervals caused by environmental conditions like the weather and wind [28,29,30,31,32,33].

**Figure 13:**
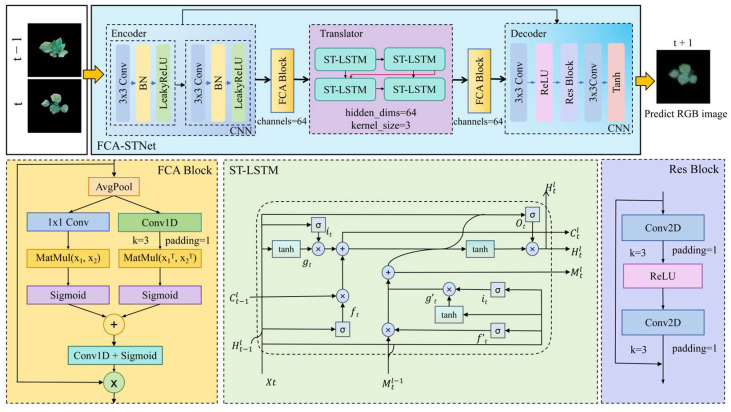
FCA-STNet network architecture.

To address these challenges, we propose FCA-STNet, a growth prediction network architecture designed to forecast images of cotton seedlings at time t + 1 using historical RGB image sequences. Built on a custom-designed STNet backbone, FCA-STNet enhances both spatiotemporal and spatial feature extraction by incorporating an Adaptive Fine-Grained Channel Attention (FCA) module. The overall network architecture is illustrated in Figure 13. FCA-STNet consists of three main components: an Encoder, Translator, and Decoder. The Encoder extracts spatial features from the input images, capturing local morphological and textural details while mitigating the effects of the lighting and occlusion. FCA is then applied to perform adaptive channel-wise weighting, suppressing redundant features. The Translator, based on ST-LSTM, models dynamic temporal dependencies within the image sequence. Another FCA module is used to reinforce the responses in key spatiotemporal channels, guiding the Decoder in constructing high-fidelity predictions of the cotton seedling’s future state at time t + 1. This structure significantly improves the prediction accuracy and robustness.

#### 4.1.1. STNet Structure

In field conditions, cotton seedling growth exhibits clear spatiotemporal evolution. Spatially, early-stage seedlings grow rapidly, yet their changes—such as slight leaf expansions or gradual height increases—are often subtle and complex, requiring high spatial sensitivity. The lighting conditions also vary, with frequent shadows, cloud cover, and overlapping vegetation leading to mixed useful and noisy information in the image, complicating spatial feature extraction. Temporally, cotton seedlings’ growth is continuous and highly dependent on the previous stages, making it difficult for static methods to capture its dynamic characteristics effectively.

**Figure 14:**
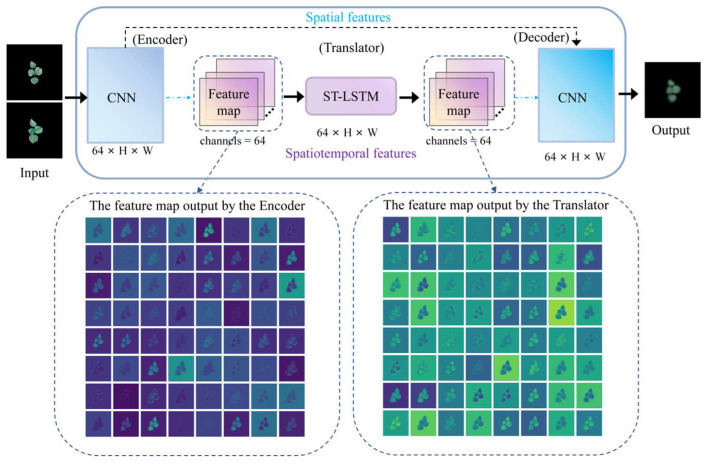
STNet architecture.

To address these issues, we improved the SimVP architecture [34] and developed STNet. The key innovation was replacing the original multi-layer Inception modules in the Translator with stacked ST-LSTM blocks and removing the upsampling operations. This redesign better accommodates the coexistence of long-term dependencies and localized variations during seedling growth, thereby improving the extraction of key spatiotemporal features. As shown in Figure 14, the modified STNet architecture consists of three primary modules: an Encoder to extract the spatial features from each frame, a Translator using ST-LSTM to model the spatiotemporal dependencies, and a Decoder to predict the image at time t + 1. This structure is particularly well-suited for forecasting plant growth with continuous dynamic changes.

Cotton seedling prediction is essentially a sequence-to-sequence image prediction task that requires accurate estimation of future frames. Seedling development is not isolated; the current states are strongly influenced by the previous growth stages. ST-LSTM is capable of capturing such temporal dependencies, which static modules like Inception cannot. Leveraging its gating mechanisms (input, forget, and output gates), ST-LSTM efficiently retains long-term dependencies, providing greater stability and robustness for long sequences. In contrast, Inception modules emphasize parallel extraction of multi-scale features and are weaker in capturing the temporal dynamics. The Translator consists of two stacked ST-LSTM layers. ST-LSTM is an enhanced version of the Long Short-Term Memory (LSTM) network [35]. Since traditional LSTM overlooks spatial correlations, Shi et al. [36] proposed ConvLSTM to incorporate both spatial and temporal features. To address the limitation of a unidirectional information flow, ST-LSTM introduces a dual-memory state mechanism. Each node at time t and layer l integrates hidden states, $H_{t}^{l}$, from two memory states,$C_{t}^{l}$ and $M_{t}^{l}$, using outputs, $O_{t}$, controlled by dual-directional signals. This facilitates deep fusion of the temporal and spatial correlations. The ST-LSTM cell operations are defined in Equations (1)–(10).

(1) $$g_{t}=\tanh(W_{xg}*X_{t}+W_{hg}*H_{t-1}^{l}+b_{g})$$

(2) $$i_{t}=\sigma (W_{xi}*X_{t}+W_{hi}*H_{t-1}^{l}+b_{i})$$

(3) $$f_{t}=\sigma (W_{xf}*X_{t}+W_{hf}*H_{t-1}^{l}+b_{f})$$

(4) $$C_{t}^{l}=f_{t}⊙C_{t-1}^{l}+i_{t}⊙g_{t}$$

(5) $$g_{t}^{\prime }=\tanh(W_{xg}^{\prime }*X_{t}+W_{mg}*M_{t}^{l-1}+b_{g}^{\prime })$$

(6) $$i_{t}^{\prime }=\sigma (W_{xi}^{\prime }*Xt+W_{mi}*M_{t}^{l-1}+b_{i}^{\prime })$$

(7) $$f_{t}^{\prime }=\sigma (W_{xf}^{\prime }*X_{t}+W_{mf}*M_{t}^{l-1}+b_{f}^{\prime })$$

(8) $$M_{t}^{l}=f_{t}^{\prime }⊙M_{t}^{l-1}+i_{t}^{\prime }⊙g_{t}^{\prime }$$

(9) $$O_{t}=\sigma (W_{xo}*X_{t}+W_{ho}*H_{t-1}^{l}+W_{co}*C_{t}^{l}+W_{mo}*M_{t}^{l}+b_{o})$$

(10) $$H_{t}^{l}=O_{t}⊙\tanh(W_{1x1}*[C_{t\prime }^{l}M_{t}^{l}])$$

In the formula, tanh() and σ() represent the tanh and Sigmoid activation functions, respectively; ∗ and ⊙ denote the convolution operator and the Hadamard product, respectively;$W_{\mathrm{xg}}$, $W_{\mathrm{hg}}$, $W_{\mathrm{xi}}$, $W_{\mathrm{hi}}$, $W_{\mathrm{xf}}$, $W_{\mathrm{hf}}$, $W_{\mathrm{xg}}^{\prime }$, $W_{\mathrm{mg}}$, $W_{\mathrm{xi}}^{\prime }$, $W_{\mathrm{mi}}$, $W_{\mathrm{xf}}^{\prime }$, $W_{\mathrm{mf}}$, $W_{\mathrm{xo}}$, $W_{\mathrm{ho}}$, $W_{\mathrm{co}}$, $W_{\mathrm{mo}}$, and $W_{1x1}$ represent the weights of the convolutions; $X_{t-1}$ represents the input at time (t − 1); $H_{t}^{l}$ represents the hidden state of the l layer at time t; $X_{t}$ is the output at time t; $C_{t-1}^{l}$ represents the cell state of layer l at time (t − 1); $C_{t}^{l}$ represents the cell state of the l layer at time t; $M_{t}^{l-1}$ represents the spatiotemporal memory of the (l−1) layer at time t; $M_{t}^{l}$ represents the spatiotemporal memory of the l layer at time t; and $b_{g}$, $b_{i}$, $b_{f}$, $b_{g}^{\prime }$, $b_{i}^{\prime }$, $b_{f}^{\prime }$, and $b_{o}$ represent the deviations.

#### 4.1.2. FCA Module

Field environments are inherently variable, often involving unpredictable weather and lighting conditions. Factors such as uneven sunlight, wind-induced leaf movement, curling, or morphological distortion can blur images, distort structures, and amplify noise, significantly impairing the extraction of texture, edge, and structural features. Although STNet possesses strong spatiotemporal feature extraction capabilities and performs well in capturing dynamic changes, it still struggles with abrupt local variations, redundant channels, and complex texture perception.

**Figure 15:**
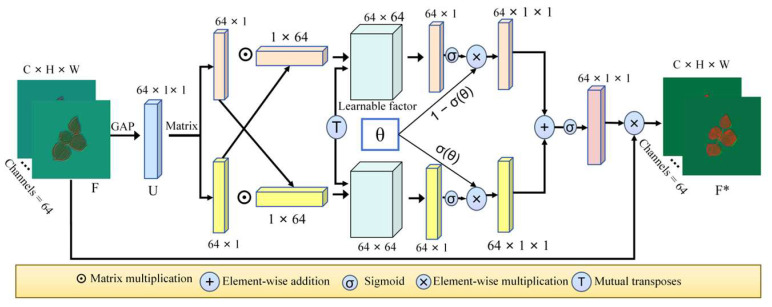
FCA module architecture.

To overcome these limitations, we integrated an Adaptive Fine-Grained Channel Attention (FCA) module [37] into STNet, forming a more selective and robust FCA-STNet architecture. FCA enhances the network’s ability to focus on stable and texture-rich regions by adaptively weighting channel features based on global contextual information. This not only suppresses noise and low-quality regions but also improves the sensitivity to informative channels. The Fine-Grained Channel Attention mechanism enables the model to better capture critical cotton features—such as the leaf texture, shape, and color distributions—thereby improving the image clarity and detail preservation. The FCA module architecture is illustrated in Figure 15.

#### 4.1.3. Evaluation Metrics

The FCA-STNet model was evaluated by comparing its predicted cotton seedling images with the ground truth images in terms of their visual similarity. Both subjective evaluation and quantitative metrics were applied, including the Structural Similarity Index (SSIM) [38], Peak Signal-to-Noise Ratio (PSNR) [39], Mean Squared Error (MSE), and Mean Absolute Error (MAE).

The SSIM evaluates the images’ similarity based on their luminance, contrast, and structural attributes. Values closer to 1 indicate greater structural similarity between the predicted and actual images.

The PSNR is calculated from the peak grayscale value and the MSE between the predicted and ground truth images. A higher PSNR indicates better image quality.

The MSE measures the average squared difference between the predicted and ground truth pixel values; lower values indicate higher prediction accuracy.

The MAE quantifies the average absolute difference in the pixel intensity; lower values suggest a reduced overall error.

The mathematical definitions of these metrics are presented as follows:

(11) $$SSIM(x,y)={\left[l(x,y)\right]}^{\alpha }\cdot {\left[c(x,y)\right]}^{\beta }\cdot {\left[s(x,y)\right]}^{\gamma }$$

(12) $$PSNR=10\cdot \mathrm{lg}(\frac{MaxValue^{2}}{MSE})$$

(13) $$MSE=\frac{1}{N}\sum_{i=1}^{N}(y_{i}-\hat{y_{i}}{)}^{2}$$

(14) $$MAE=\frac{1}{N}\sum_{i=1}^{N}|y_{i}-\hat{y_{i}}|$$

In the formula, l(x,y) represents the brightness comparison; c(x,y) represents the contrast comparison; s(x,y) represents the structure comparison; x and y, respectively, denote the two images or local regions to be compared; α, β, and γ are weight parameters, usually set to 1; MaxValue is the maximum value that the image pixels can have; N is the number of test sample sets; $y_{i}$ is the true value; and $\hat{y_{i}}$ is the predicted value.

### 4.2. Phenotypic Feature Extraction

**Figure 16:**
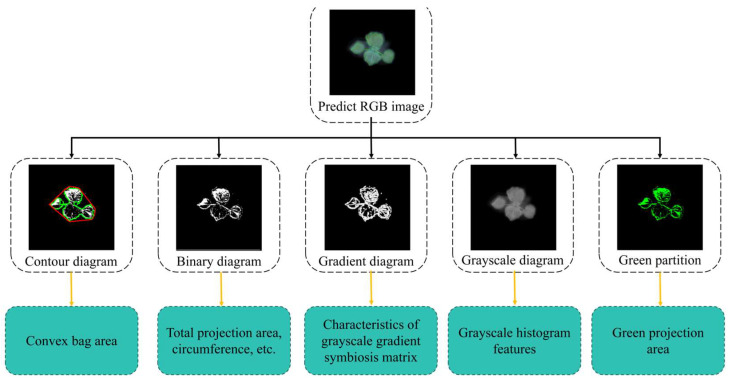
Phenotypic feature extraction workflow. Black lines indicate image processing steps; orange lines indicate parameter extraction steps.

Phenotypic features were extracted from the images of the cotton seedlings’ predicted appearance at time t + 1 using FCA-STNet. As shown in Figure 16, both ground truth and predicted images from the test set were analyzed for their texture, morphological, and color-related traits. The Pearson correlation coefficient [40] was used to quantify the linear relationships between the predicted and true features. A value closer to 1 indicates a strong positive correlation and reflects the model’s accuracy in phenotypic prediction.

Using OpenCV, we extracted a total of 37 phenotypic traits: 21 texture features, 10 morphological features, and 6 color features. The feature extraction workflow is illustrated in Figure 16. The predicted RGB image is first converted into a binary mask from which contour features such as the perimeter and bounding rectangle are extracted. The convex hull area is derived from the contour mask. The RGB image is also converted into an HSL color space, and an H-channel gradient image is computed. From this gradient image, the gray-level co-occurrence matrix (GLCM) and histogram features are extracted. Finally, the red, green, and blue projection areas are computed using color thresholding.

## 5. Conclusions

To address the limitations of low visual realism and insufficient texture feature extraction in the prediction of future cotton seedling growth images under field conditions, this study proposes the FCA-STNet growth prediction model. By leveraging historical image sequences of cotton seedling development, the model forecasts future-stage growth images and enables downstream phenotypic feature extraction based on the predicted outputs. The experimental results demonstrate that the model can generate predictions with high visual fidelity. Moreover, the quantitative metrics—the MSE, MAE, SSIM, and PSNR—reached 0.0086, 0.0321, 0.8339, and 20.7011, respectively, representing improvements over the baseline STNet by 2.27%, 0.31%, 4.73%, and 11.20%. The proposed model also outperforms several mainstream spatiotemporal prediction frameworks.

In addition, a total of 37 phenotypic features—encompassing texture, morphological, and color traits—were extracted from both predicted and ground truth images of the seedlings at future time points. Pearson correlation analysis revealed that the model achieved excellent performance in predicting the texture and morphological features. Notably, the correlation coefficients for traits such as the Mean Gray Value (M: 0.96), Uniformity (U: 0.91), Entropy (E: 0.90), Total Projected Area (TPA: 0.91), and Average Minimum Bounding Area (A_MBA: 0.95) all exceeded 0.9, indicating strong consistency and stability. However, limitations remain in the model’s ability to predict certain traits. For instance, the color feature Red Projected Area (RPA) yielded a low correlation coefficient of 0.07, and some morphological features—such as the Circularity (R: 0.65) and Rotation Angle (AN: 0.39)—also exhibited suboptimal predictive performance. These areas warrant further investigation and methodological refinement in future work.

## Figures and Tables

**Table 1:** Evaluation metrics for different prediction models.

Model	MSE↓	MAE↓	SSIM↑	PSNR↑
PredRNN	0.0188	0.1205	0.0233	16.2197
PredRNN++	0.0184	0.0900	0.2660	3.0651
MIM	0.0107	0.0423	0.5435	20.0066
ConvLSTM	0.0091	0.0351	0.6038	18.5990
SwinLSTM	0.0099	0.0361	0.7873	20.3337
ConvGRU	0.0085	0.0326	0.7994	18.0765
TrajGRU	0.0084	0.0313	0.8115	13.3757
SimVP	0.0137	0.0555	0.8114	20.1878
FCA-STNet (Ours)	0.0086	0.0321	0.8339	20.7011

**Table 2:** FCA-STNet ablation experiment.

STNet	FCA		MSE↓	MAE↓	SSIM↑	PSNR↑
	Encoder	Translator				
√			0.0088	0.0322	0.7962	18.6170
√	√		0.0086	0.0321	0.7993	19.7616
√		√	0.0089	0.0318	0.7704	15.8190
√	√	√	0.0086	0.0321	0.8339	20.7011

**Table 3:** Table comparing and evaluating different parameters, layers, and models used in Translator.

Translator	Layers			hidden_dims			MSE↓	MAE↓	SSIM↑	PSNR↑
	1	2	3	32	64	128				
ST-LSTM	√			√			0.0084	0.0311	0.7211	15.5391
	√				√		0.0084	0.0299	0.7946	16.5742
	√					√	0.0080	0.0315	0.7260	16.6728
		√			√		0.0088	0.0322	0.7962	18.6170
			√		√		0.0084	0.0340	0.6888	17.8139
ConvLSTM		√			√		0.0082	0.0324	0.7248	18.8963
ConvGRU		√			√		0.0085	0.0308	0.7802	16.6416

**Table 4:** Table showing quantitative evaluation of different attention mechanisms’ performance.

Name of the Attention Mechanism	MSE↓	MAE↓	SSIM↑	PSNR↑
FCA	0.0086	0.0321	0.8339	20.7011
CGA	0.0089	0.0411	0.0123	0.95110
FSAS	0.0083	0.0338	0.6591	19.8130
EPA	0.0092	0.0321	0.6921	15.0228

**Table 5:** Coefficients of correlations between predicted and actual phenotypes.

Trait Category	Trait Type	Abbreviation	Definition	PCC
Texture Features	Gray Histogram	M	Mean	0.96
		SE	Smoothness	0.87
		S	Standard Deviation	0.88
		MU3	Third-Order Moment	0.74
		U	Uniformity	0.91
		E	Entropy	0.90
	GLCM Features	T1	Correlation	0.70
		T2	Low-Gradient Emphasis	0.92
		T3	High-Gradient Emphasis	0.65
		T4	Energy	0.91
		T5	Gray-Level Non-Uniformity	0.91
		T6	Gradient Non-Uniformity	0.91
		T7	Gray Mean	0.96
		T8	Gradient Mean	0.96
		T9	Gray Entropy	0.90
		T10	Gradient Entropy	0.90
		T11	Mixed Entropy	0.91
		T12	Difference Moment	0.65
		T13	Inverse Difference Moment	0.92
		T14	Gray Standard Deviation	0.91
		T15	Gradient Standard Deviation	0.91
Morphological Features	Overall Morphology	TPA	Total Projected Area	0.91
		A_HA	Convex Hull Area	0.95
		R	Circularity	0.65
		AN	Rotation Angle	0.39
	Average Leaf Shape	A_A	Average Leaf Area	0.90
		A_P	Average Leaf Perimeter	0.89
		A_MBA	Average Minimum Bounding Box Area	0.95
	Ratio-Based Morphology	A_TBR	Ratio of Average Total Area to Bounding Box	0.71
		A_TMBR	Ratio of Average Total Area to Min Box	0.75
		A_PAR	Perimeter-to-Area Ratio	0.77
Color Features		RPA	Red Projected Area	0.07
		GPA	Green Projected Area	0.67
		BPA	Blue Projected Area	0.60
		RTR	Ratio of Red Area to Total Projected Area	0.06
		GTR	Ratio of Green Area to Total Projected Area	0.15
		BTR	Ratio of Blue Area to Total Projected Area	0.19

## Data Availability

The data presented in this study are available on request from the corresponding author. The data are not publicly available for privacy reasons.

## References

[1] Zhao C.J., Lu S.L., Guo X.Y., Xiao B.X., Wen W.L. (2010). Exploration of digital plants and their technical system. Sci. Agric. Sin..

[2] Zhang H.C., Wang G.S., Bian L.M., Zheng J.Q., Zhou H.P. (2019). Research on plant phenotype measurement system and temporal growth model based on optical cameras. Trans. Chin. Soc. Agric. Mach..

[3] Zhu X.G., Chang T.G., Song Q.F., Chang S.Q., Wang C.R., Zhang G.Q., Guo Y., Zhou S.C. (2020). Digital plants: Scientific connotation, bottlenecks, and development strategies. Syn. Biol. J..

[4] Espana M., Baret F., Aries F., Chelle M., Andrieu B., Prevot L. (1999). Modeling maize canopy 3D architecture: Application to reflectance simulation. Ecol. Model..

[5] Qian B., Huang W.J., Xie D.H., Ye H.C., Guo A., Pan Y.H., Jin Y., Xie Q.Y., Jiao Q.J., Zhang B.Y. (2023). Coupled maize model: A 4D maize growth model based on growing degree days. Comput. Electron. Agric..

[6] Minorsky P.V. (2003). Achieving the in silico plant: Systems biology and the future of plant biological research. Plant Physiol..

[7] Prusinkiewicz P. (2004). Modeling plant growth and development. Curr. Opin. Plant Biol..

[8] Kim T.H., Lee S.H., Oh M.M., Kim J.O. Plant growth prediction based on hierarchical auto-encoder. Proceedings of the International Conference on Electronics, Information and Communication (ICEIC).

[9] Kim T.H., Lee S.H., Kim J.O. (2022). A novel shape-based plant growth prediction algorithm using deep learning and spatial transformation. IEEE Access.

[10] Sakurai S., Uchiyama H., Shimada A., Taniguchi R. Plant growth prediction using convolutional LSTM. Proceedings of the 14th International Joint Conference, VISIGRAPP 2019.

[11] Wang C.Y., Pan W.T., Li X., Liu P. (2022). A plant growth and development prediction model based on STLSTM. Trans. Chin. Soc. Agric. Mach..

[12] Wang C., Pan W., Song X., Yu H., Zhu J., Liu P., Li X. (2022). Predicting plant growth and development using time-series images. Agronomy.

[13] Yasrab R., Zhang J., Smyth P., Pound M.P. (2021). Predicting plant growth from time-series data using deep learning. Remote Sens..

[14] Drees L., Junker-Frohn L.V., Kierdorf J., Roscher R. (2021). Temporal prediction and evaluation of Brassica growth in the field using conditional generative adversarial networks. Comput. Electron. Agric..

[15] Duan L.F., Wang X.Y., Wang Z.H., Geng Z.D., Lu Y.R., Yang W.N. (2024). A visualization prediction method of multi-variety rice growth based on improved Pix2Pix-HD network. Acta Agron. Sin..

[16] Snider J.L., Collins G.D., Whitaker J.R., Chapman K.D., Horn P.J. (2016). The impact of seed size and chemical composition on seedling vigor, yield, and fiber quality of cotton in five production environments. Field Crops Res..

[17] Shi X., Gao Z., Lausen L., Wang H., Yeung D.Y., Wong W.K., Woo W.C. Deep learning for precipitation nowcasting: A benchmark and a new model. Proceedings of the Advances in Neural Information Processing Systems (NeurIPS).

[18] Tang S., Li C., Zhang P., Tang R. SwinLSTM: Improving spatiotemporal prediction accuracy using Swin Transformer and LSTM. Proceedings of the 2023 IEEE/CVF International Conference on Computer Vision (ICCV).

[19] Kong L., Dong J., Li M., Ge J., Pan J.S. Efficient frequency domain-based transformers for high-quality image deblurring. Proceedings of the 2023 IEEE/CVF Conference on Computer Vision and Pattern Recognition (CVPR).

[20] Lu L., Xiong Q., Chu D., Xu B. MixDehazeNet: Mix structure block for image dehazing network. Proceedings of the 2024 International Joint Conference on Neural Networks (IJCNN).

[21] Wang Y., Zhang J., Zhu H., Long M., Wang J., Yu P.S. Memory in memory: A predictive neural network for learning higher-order non-stationarity from spatiotemporal dynamics. Proceedings of the 2019 IEEE/CVF Conference on Computer Vision and Pattern Recognition (CVPR).

[22] Wang Y., Wu H., Zhang J., Gao Z., Wang J., Yu P.S., Long M. (2021). PredRNN: A recurrent neural network for spatiotemporal predictive learning. IEEE Trans. Pattern Anal. Mach. Intell..

[23] Wang Y., Gao Z., Long M., Wang J., Yu P.S. PredRNN++: Towards a resolution of the deep-in-time dilemma in spatiotemporal predictive learning. Proceedings of the International Conference on Machine Learning (ICML).

[24] Ronneberger O., Fischer P., Brox T. (2015). U-Net: Convolutional networks for biomedical image segmentation. arXiv.

[25] Krizhevsky A., Sutskever I., Hinton G.E. (2012). ImageNet classification with deep convolutional neural networks. Commun. ACM.

[26] Siam M., Valipour S., Jägersand M., Ray N. Convolutional gated recurrent networks for video segmentation. Proceedings of the 2017 IEEE International Conference on Image Processing (ICIP).

[27] Chen Z., He Z., Lu Z.M. (2023). DEA-Net: Single image dehazing based on detail-enhanced convolution and content-guided attention. IEEE Trans. Image Process..

[28] Reddy K.R., Brand D., Wijewardana C., Gao W. (2017). Temperature effects on cotton seedling emergence, growth, and development. Agron. J..

[29] Wanjura D.F., Buxton D.R. (1972). Hypocotyl and radicle elongation of cotton as affected by soil environment. Agron. J..

[30] Steiner J.J., Jacobsen T.A. (1992). Time of planting and diurnal soil temperature effects on cotton seedling field emergence and rate of development. Crop Sci..

[31] Liu R.S. (2011). Effects of density on cotton seedling transplanting in southern Xinjiang. J. Anhui Agric. Sci..

[32] Tamadon-Rastegar M., Gharineh M.H., Mashadi A.A., Siadat S.A., Barzali M. (2015). The effects of moisture stress on seedling growth characteristics of cotton cultivars. Int. J. Agric. Biosci..

[33] Oosterhuis D.M., Miley W.N., Oosterhuis D.M. (1990). Growth and development of a cotton plant. Nitrogen Nutrition of Cotton.

[34] Gao Z., Tan C., Wu L., Li S.Z. SimVP: Simpler yet better video prediction. Proceedings of the 2022 IEEE/CVF Conference on Computer Vision and Pattern Recognition (CVPR).

[35] Wang Y.B., Long M.S., Wang J.M., Gao Z., Yu P.S. PredRNN: Recurrent neural networks for predictive learning using spatiotemporal LSTMs. Proceedings of the 31st International Conference on Neural Information Processing Systems (NeurIPS).

[36] Shi X.J., Chen Z.R., Wang H., Yeung D.Y., Wong W.K., Woo W.C. Convolutional LSTM network: A machine learning approach for precipitation nowcasting. Proceedings of the 28th International Conference on Neural Information Processing Systems (NeurIPS).

[37] Sun H., Wen Y., Feng H., Zheng Y., Mei Q., Ren D., Yu M. (2024). Unsupervised bidirectional contrastive reconstruction and adaptive fine-grained channel attention networks for image dehazing. Neural Netw..

[38] Chen Y., Liu L., Phonevilay V., Gu K., Xia R., Xie J., Zhang Q., Yang K. (2021). Image super-resolution reconstruction based on feature map attention mechanism. Appl. Intell..

[39] Pearson K. (1896). Mathematical contributions to the theory of evolution. III. Regression, heredity, and panmixia. Philos. Trans. R. Soc. A.

[40] Oh J., Guo X., Lee H., Lewis R.L., Singh S. Action-conditional video prediction using deep networks in Atari games. Proceedings of the Advances in Neural Information Processing Systems (NeurIPS).

